# Profiling Individual Differences in Alcoholic Beverage Preference and Consumption: New Insights from a Large-Scale Study

**DOI:** 10.3390/foods9081131

**Published:** 2020-08-17

**Authors:** Maria Carla Cravero, Monica Laureati, Sara Spinelli, Federica Bonello, Erminio Monteleone, Cristina Proserpio, Maria Rosa Lottero, Ella Pagliarini, Caterina Dinnella

**Affiliations:** 1 Council for Agricultural Research and Economics (CREA), Research Centre for Viticulture and Enology, via Pietro Micca 35, 14100 Asti, Italy; mariacarla.cravero@crea.gov.it (M.C.C.); federica.bonello@crea.gov.it (F.B.); mariarosa.lottero@crea.gov.it (M.R.L.); 2 Department of Food Environmental and Nutritional Sciences (DeFENS), University of Milan, Via Celoria 2, 20133 Milan, Italy; cristina.proserpio@unimi.it (C.P.); ella.pagliarini@unimi.it (E.P.); 3 Department of Agriculture, Food, Environment and Forestry (DAGRI), University of Florence, via Donizetti 6, 50144 Florence, Italy; erminio.monteleone@unifi.it (E.M.); caterina.dinnella@unifi.it (C.D.)

**Keywords:** wine, beer, spirits, cocktails, age, gender, PROP, taste responsiveness, consumer segmentation

## Abstract

Alcoholic beverage consumption plays an important role in European culture, and in many contexts drinking alcohol is socially acceptable and considered part of the diet. Understanding the determinants of alcohol preference and consumption is important not only for disease prevention, intervention, and policy management, but also for market segmentation, product development, and optimization. The aim of the study was to investigate the effect of individual responsiveness to various oral sensations on self-reported liking and intake of 14 alcoholic and non-alcoholic beverages (including beers, wines, spirits, and cocktails) considering gender, age, and oral responsiveness (measured through response to 6-*n*-propyl-2-thiouracil -PROP, basic tastes, astringency, and pungency) in a large sample of Italian consumers. Data were collected from 2388 respondents (age range 18–60 years; mean age = 37.6, SD = 13.1; 58.2% women). These results indicate that notwithstanding the strong gender difference, with women generally liking and consuming fewer alcoholic beverages than men, liking patterns in the two genders were similar. Three liking patterns for different alcoholic beverages largely driven by orosensory properties were identified in both genders. “Spirit-lovers” constituted the smallest group (12%), consumed alcoholic beverages of any kind (not only spirits) more than the other segments, and were mainly men aged 30–45. “Beer/wine lovers” (44%) were the oldest group with no difference by gender. “Mild-drink lovers” (44%) liked alcoholic drinks with intense sweet taste and/or mixers that moderate ethanol perception. They were mainly women, aged 18–29, had a lower consumption of alcohol, and a higher orosensory responsiveness than the other two groups. The results also suggest the opportunity to develop personalized recommendations towards specific consumer segments based not only on socio-demographics but considering also perceptive variables. Finally, our data suggest that increased burning and bitterness from alcohol may act as a sensory hindrance to alcoholic beverage overconsumption.

## 1. Introduction

Alcoholic beverage consumption plays an important role in European culture, and in many contexts drinking alcohol is socially acceptable and considered part of the diet [1]. According to data from the World Health Organization (WHO), Europe is the heaviest drinking area worldwide, consuming about a quarter of total alcohol [2]. The Italian population alcohol consumption per capita (year 2010), expressed in L of pure alcohol, corresponded on average to 9.9 L, 11.9 L for men and 7.2 for women, with the highest consumption corresponding to wine (66%), beer (23%), and spirits (11%) [2].

Many factors influence alcohol intake, including genetics, psychological conditions, social and cultural norms, socio-demographics, and taste responsiveness [3,4]. A better understanding of the determinants of alcoholic beverage preference and consumption is important not only for disease prevention, intervention, and policy management, but also for market segmentation, product development, and optimization [4].

The most robust result in research on alcohol behavior is related to the gender gap in the frequency and amount of alcohol consumed, with men drinking more alcohol and more frequently than women [5,6]. Considering age, results are less consistent; however, in general, a trend for younger people to drink alcohol less frequently but in larger amounts and for older people smaller amounts of alcohol more frequently was reported [2].

Oral responsiveness to taste and mouthfeel sensations has also been shown to influence alcohol consumption [4]. Alcohol elicits multiple and complex oral sensations spanning from sweetness, bitterness, astringency, and a set of trigeminal sensations such as stinging, tingling, irritation, and burning [7]. Considering the substantial individual variation in sensory perception, oral sensation responsiveness may reasonably account for differences in alcohol consumption behavior [8].

The association between oral responsiveness and alcohol consumption has been investigated mainly in relation to health issues including addiction. A number of studies have focused on alcohol consumption and markers of genetic variation in taste responsiveness, i.e., the ability to taste the bitterness of 6-*n*-propyl-2-thiouracil (PROP) or phenylthiocarbamide (PTC). In general, findings indicate that individuals who taste PROP as intensely bitter also taste alcohol as more bitter and more irritating; these alcohol nociceptive sensations may act as a deterrent to alcohol consumption [9]. More specifically, Duffy et al. [10,11] found that PROP non-tasters perceived less intensely bitterness and oral burn from alcohol and were more likely to have greater preference for [11] and consumption of alcohol [10,11]. Intranuovo and Powers [12] reported that those least able to taste PROP bitterness consumed a greater number of beers than those who tasted PROP as intensely bitter. Regular consumers of beer were also more likely to be non-tasters of PROP/PTC than low consumers according to findings by Guinard et al. [13]. This study further found that low consumption of beer was driven by bitter taste disliking. Thibodeau et al. [8] evidenced that regardless of the type of oral sensation, the most responsive subjects tended to drink fewer alcoholic beverages. In a large-scale study, Fischer et al. [14] found that consumers of alcoholic beverages perceived salt, sweet, and sour to be significantly less intense than non-drinkers, but after adjusting for age and gender the relation was significant only for salt and sweet.

Although a number of studies has been conducted to explore the association between oral perception and alcohol consumption, several limitations of the existing literature have been highlighted [4]. In general, almost all studies involved a reduced number of volunteers (≈30–200), who were often selected from a non-representative sample of the population (e.g., convenience sample of college students) making it difficult to generalize the results. When large-scale studies were conducted, only a segment of the population was involved (>40 years) [15], a single alcoholic beverage category (i.e., wine) was investigated [16] or alcohol consumption was evaluated globally without detailed information regarding the specific beverages [14].

In view of these limitations, the aim of this study was to investigate the effect of individual responsiveness to various oral sensations (bitterness from PROP and caffeine, sweetness, sourness, saltiness, astringency, and pungency) on preference and consumption of 14 alcoholic and non-alcoholic beverages (including beers, wines, spirits, and cocktails) in a large sample of consumers recruited in various regions of Italy. Age and gender effects on alcohol preference and consumption were also investigated since these factors are known to influence both alcohol behavior and taste responsiveness. Finally, we explored and characterized segments of consumers varying in alcoholic preferences separated by gender (within-gender approach).

## 2. Materials and Methods

The present paper is part of the Italian taste (IT) project, a large-scale study aimed at exploring the associations among biological, genetic, physiological, sociocultural, psychological, and personality-related factors, describing the dimensions of food liking, preference, behavior, and choice, and their relevance in determining individual differences within a given food culture framework [17].

The IT project started in 2014 within the Italian Sensory Science Society (SISS) involving, on a voluntary basis, 58 SISS members working in 19 sensory laboratories of public and private organizations across the country. This study consisted of an online questionnaire session (at home) and a one-on-one testing in a sensory laboratory across 2 days. Only a selection of these tests will be presented here. For a complete overview of the study and further details on the definition of the procedures, see Monteleone et al. [17].

### 2.1. Participants

Data were collected from 2388 subjects (age range 18–60 years; mean age = 37.6, SD = 13.1; 58.2% women). The mean age of men and women was37.8 years (SD = 12.9) and 37.5 years (SD = 13.3), respectively. In order to explore possible age-related differences, respondents were divided in three age groups: 18–29 years (38.3%), 30–45 years (28.4%), 46–60 years (33.3%). The exclusion criteria were pregnancy and not being born in Italy. Participants recruitment details are reported in Monteleone et al. [17]. Briefly, participants were recruited on a national basis by means of announcements published on social networks (Facebook), articles published in national newspapers, and in magazines. Furthermore, each research unit recruited subjects locally by means of social networks, mailing lists, pamphlet distribution, and word of mouth.

The study was conducted in agreement with the Italian ethical requirements on research activities and personal data protection (D.L. 30.6.03 n. 196). The study protocol was approved by the Ethics Committee of Trieste University. The respondents gave their written informed consent at the beginning of the test according to the principles of the Declaration of Helsinki.

### 2.2. Procedure

#### 2.2.1. Assessment of Alcoholic Beverage Consumption and Liking

Prior to the laboratory sessions, respondents completed at home an online questionnaire in order to collect information about their age, gender, country of birth, and their consumption of alcoholic beverages, such as beer (*“On average, how many glasses/cans of beer do you drink per week?”*), wine *(“On average, how many glasses of wine do you drink per week?”*), liquors/spirits (*“On average, how many times a week do you consume liquor or spirits?”*), aperitifs/cocktails (*“On average, how many times a week do you drink alcoholic beverages as aperitifs, liqueur wines, cocktails?”*). Answers were provided as number of glasses/cans/times of consumed drink. The different phrasing for the different alcoholic beverages was due to the fact that in Italy there is not a standard portion (e.g., glass or can) of spirits and cocktails, which can actually be consumed in very different formats. Respondents who declared zero weekly consumption of all four types of alcoholic beverages were defined as occasional drinkers (ODs). In fact, they cannot be defined strictly as abstainers or non-drinkers as many of them, while reporting to not consume alcohol in a limited time frame (in a week), expressed a liking rating for various alcoholic beverages (see below). Therefore, we assumed they were people that probably were used to drink alcohol only in special occasions (e.g., celebrations).

Moreover, participants were asked to report their liking for 14 alcoholic and non-alcoholic drinks (blonde beer, dark beer, dry spumante, sweet spumante, red wine, white wine, sparkling white wine, limoncello, grappa, liquors, passito wine, whisky, alcoholic and non-alcoholic aperitifs) using a 9-point hedonic scale [18] anchored at the extremes: 1 = “extremely disliked” and 9 = “extremely liked”, using as middle point of the scale 5 = “neither liked nor disliked”. These drinks were selected in order to have a wide representation of the different alcoholic beverages and cocktails most frequently consumed in Italy. If the participant had never tasted the product in question, she/he could choose the answer, “I have never tasted it”, and these were treated as missing values. These respondents will be referred to as “never tasted” along the text.

#### 2.2.2. Assessment of Chemosensory Responsiveness

##### Responsiveness to Basic Tastes, Astringency, and Pungency

Participants were invited to take part in a tasting session in a sensory laboratory setting and were asked to rate the intensity of six water solutions representing four basic tastes and the sensations of astringency and pungency. The intensity of all water solutions was assessed using the generalized labeled magnitude scale, gLMS (0–100) [19]. The concentration of the solutions was decided based on published psychophysical data [20,21,22] and preliminary trials conducted with one hundred untrained subjects (unpublished data) in order to select solutions equivalent to moderate/strong on a gLMS (sourness: Citric acid 4 g/kg; bitterness: Caffeine 3 g/kg; sweetness: Sucrose 200 g/kg; saltiness: Sodium chloride 15 g/kg; astringency: Potassium aluminum sulfate 0.8 g/kg; pungency: Capsaicin 1.5 mg/kg) [17].

Participants were trained in the use of the gLMS consistent with standard procedures [23,24,25]. The gLMS consisted of a 100-unit vertical line with labels placed at no sensation, 0; barely detectable, 1.4; weak, 6; moderate, 17; strong, 34.7; very strong, 52.5; and the strongest imaginable sensation of any kind, 100. Numerical labels were not reported on the scale. Subjects were instructed to think to the “strongest imaginable sensation” as the most intense sensation they could imagine that involves remembered/imagined sensations in any sensory modality. This included such varied sensations as the cold of a cube of ice in the mouth, the deafening noise of a plane that is flying low, the strong pain felt when shutting a finger in a door. Participants were invited to ask questions about how to use the scale, and a practice trial was given in which they were asked to evaluate the intensity of the most intense light they have ever experienced using a paper version of the scale.

Respondents evaluated the solutions in individual booths under normal light conditions and were informed about the sensory quality that they were tasting. Water solutions were presented simultaneously (10 mL) in 80-mL plastic cups identified with a random three-digit code. The presentation order of the samples was randomized, except for the capsaicin solution, which was always presented last to minimize carry-over effects. Participants were instructed to hold the sample in their mouth for 3 s, then expectorate, wait 3 s (5 s in the case of bitterness, astringency, and burning) and evaluate the intensity of the sensation on the gLMS. After each sample, participants rinsed their mouth with water and waited 60 s before evaluating the following sample. After the capsaicin solution, participants rinsed their mouth with water, had a cracker, and rinsed again with water for at least 180 s before proceeding to the following test.

##### Responsiveness to PROP

In a separate session, participants also rated the intensity of a water solution of 6-*n*-propyl-2-thiouracil (PROP). A supra-threshold 3.2 mM solution was prepared by dissolving 0.5447 g/L of PROP (European Pharmacopoeia Reference Standard, Sigma Aldrich, Milano, IT) into deionized water [26]. Subjects were presented with two identical samples (10 mL) in plastic cups, coded with three-digit numbers [22]. Subjects were instructed to hold each sample (10 mL) in their mouth for 10 s, then expectorate, wait 20 s, and evaluate the intensity of bitterness using the gLMS [19]. Subjects had a 90 s break in order to control for carry-over effect after the first sample evaluation. During the break, subjects rinsed their mouth with water for 30 s, had some plain crackers for 30 s, and finally rinsed with water for a further 30 s. The average bitterness score was used for each subject.

### 2.3. Data Analysis

Two-way analyses of variance (ANOVAs) were performed to investigate the effect of age (18–29 years, 30–45 years, 46–60 years), gender (men, women), and their interaction on alcoholic beverage consumption (beer, wine, liquors/spirits, and aperitif/cocktails) and liking (14 different drinks). When the ANOVAs showed a significant effect (*p* < 0.05), post-hoc comparisons using the Bonferroni test adjusted for multiple comparison were used.

In order to explore segments of consumers varying in alcoholic beverage preference, a principal component analysis (PCA) was computed on liking data of the 14 beverages. The analysis was conducted separately for women and men (within-gender approach). Initially, the PCA was performed by removing the ODs as it was believed that these subjects might be less representative of the preference for alcoholic beverages. However, the plots that were obtained without ODs were very similar to those of the total sample. We assumed that this similarity was due to the fact that, despite having a low alcohol consumption, ODs still have a well-defined preference for alcoholic beverages. For this reason, we identified liking patterns for different alcoholic beverages including ODs. Data were standardized (i.e., scaled to unit variance) prior to modeling and full cross validation was chosen as validation method. Consumers with more than 50% of missing values (*n* = 57, which corresponded to most of the “never tasted” subjects) were automatically removed from the analysis. The PCAs were thus run on a total of 2331 subjects (1353 women and 978 men). Based on loadings coordinates on the first two principal components (PC1, PC2), subjects were grouped into three segments, representing liking patterns for different alcoholic beverages (see details in Section 3.3). The three consumer liking segments were inspected for alcoholic beverage consumption and for chemosensory responsiveness. First, a 2-way ANOVA was run considering age (3 age groups), consumer segments based on liking (three segments), and their interaction as factors. After verifying that the contribution of age was marginal and did not provide further information compared to the models considered previously, differences in alcoholic beverage consumption and in chemosensory responsiveness between the three liking segments were investigated through 1-way ANOVA followed by the Bonferroni post-hoc test considering consumer segments based on liking (three segments) as factor. Differences related to drinking behavior, age, and gender among consumer segments were analyzed using a chi-square test.

The SAS/STAT statistical software package version 9.4 (SAS Institute Inc., Cary, NC, USA) and the Unscrambler software version 11.0 (CAMO Software AS, Oslo, Norway) were used for the data analysis.

## 3. Results

### 3.1. Alcoholic Beverage Consumption

#### 3.1.1. Age and Gender Differences

On average, mean consumption of beer and wine corresponded to 1.3 and 2.4 glasses per week, respectively. Spirits/liquors and aperitifs/cocktails were consumed 0.4 and 0.7 times per week, respectively. Four hundred and eighty-nine subjects (20.5% of the total sample) stated a zero weekly average consumption of alcoholic beverages (occasional drinkers, ODs). Among the ODs, 77% were women, whereas similar OD proportions were found across age groups (18–29 years = 31%; 30–45 years = 34%; 46–60 years = 35%).

There were strong effects of gender and age on alcoholic beverage consumption (Table 1).

Gender was highly significant for all tested beverages (*p* < 0.0001), with women consuming fewer alcoholic beverages than men. Women consumed mainly wine, followed by beer, aperitifs/cocktails, and lastly spirits/liquors. Similarly, men consumed mainly wine, followed by beer, but had a similar consumption of aperitifs/cocktails and spirits/liquors.

Age was also highly significant for all tested beverages (*p* < 0.0001). Frequency of consumption of all alcoholic beverages systematically decreased with increasing age, with the exception of wine, whose consumption increased with age. Wine consumption was also influenced by the interaction gender × age (*p* < 0.0001). Specifically, wine consumption increased significantly with age in men, whereas in women the trend was not significant. None of the other interactions were significant.

#### 3.1.2. Association with Chemosensory Perception

Although correlation coefficients were low, the intake of all alcoholic drinks was negatively but significantly associated with responsiveness to pungency (for beer, wine, and spirits: r coefficients ranged from −0.10 to −0.12, *p* < 0.0001; for cocktails: r = −0.06, *p* < 0.001). PROP responsiveness correlated negatively with beer (r = −0.09, *p* < 0.0001), wine (r = −0.08, *p* < 0.0001), and spirit intake (r = −0.05, *p* < 0.05).

### 3.2. Alcoholic Beverage Liking

In general, the proportion of subjects stating they had never tasted the beverage in question (“never tasted” subjects) was small, ranging from 1.2% (sweet spumante) to 7.1% (Grappa), and only in the case of passito wine and whisky was higher than 10% (11.4% and 12.8%, respectively). In almost all cases, the percentage of women declaring having never tasted a certain drink was significantly higher than that of men. Mean liking scores calculated for the overall population (*n* = 2388) by age and gender are reported in Table 2.

Strong effects of age and gender on alcoholic beverage liking were found. Gender affected the liking of all beverages, except sweet spumante, with women liking alcohol less than men.

Age also influenced the liking of almost all beverages with the exception of beers, white and sparkling white wines, and limoncello. With increasing age, acceptability of dry spumante, red wine, passito wine, and grappa increased, whereas a decrease was seen for sweet spumante, liquors, whisky, and both aperitifs.

Liking for red wine, limoncello, and alcoholic aperitif was affected by the interaction gender × age (*p* < 0.05 in all cases). Liking for red wine and limoncello decreased with age for men but not for women, whereas liking for alcoholic aperitifs decreased with increasing age in both genders, but the decrease was steeper for women than men.

### 3.3. Identification of Liking Patterns for Different Alcoholic Beverages

**Figure 1:**
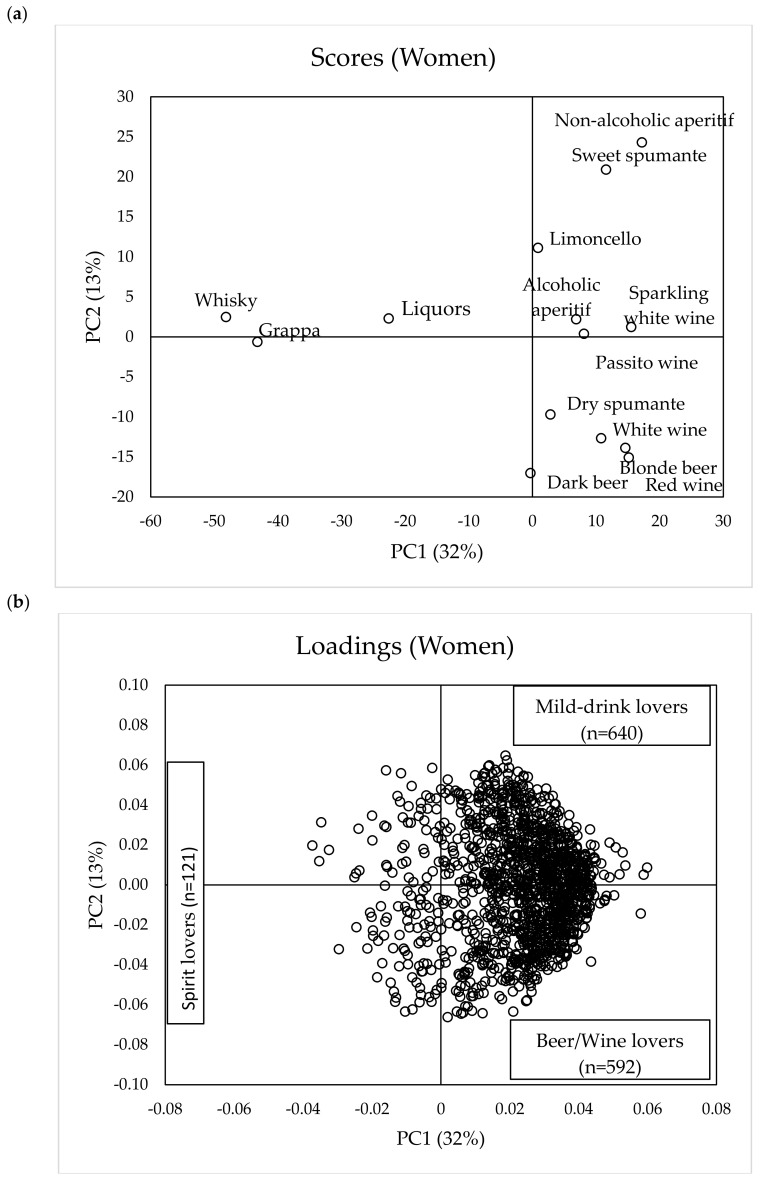
Scores (**a**) and loadings (**b**) obtained from principal component analysis (PCA) performed on liking (9-point hedonic scale) of different alcoholic beverages by women consumers (*n* = 1353).

**Figure 2:**
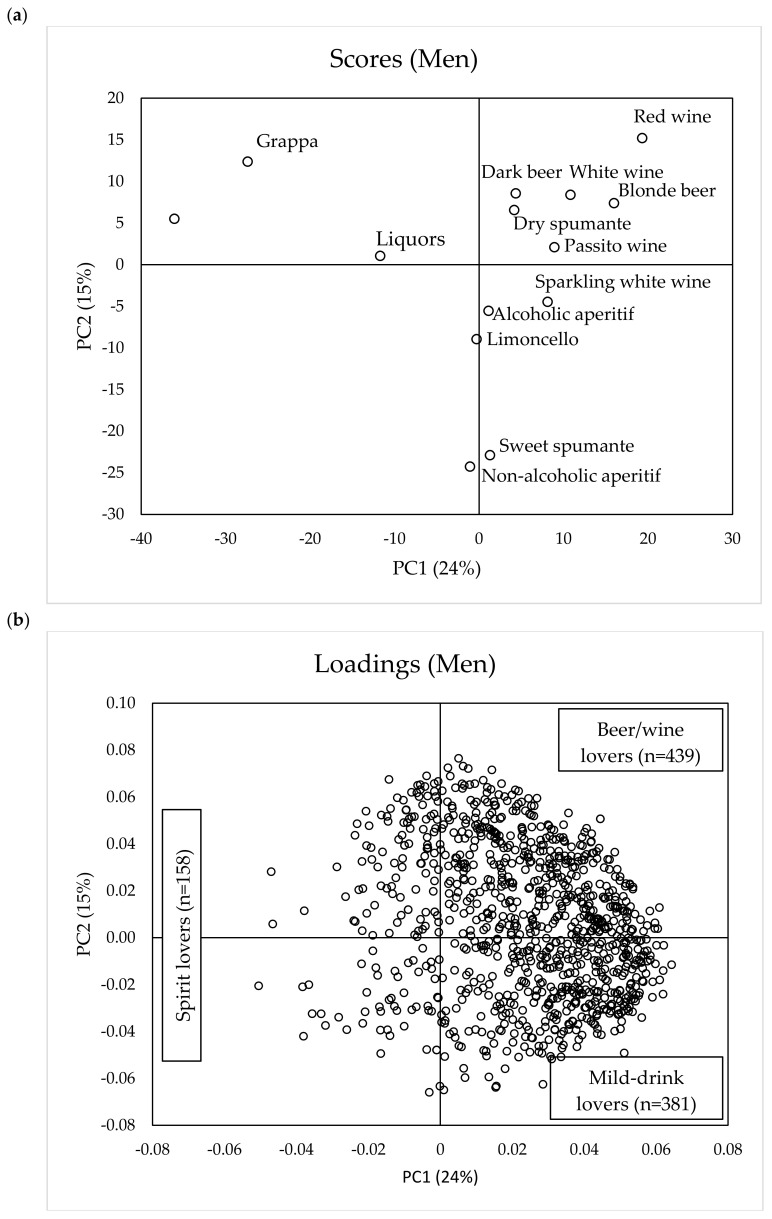
Scores (**a**) and loadings (**b**) obtained from PCA performed on liking (9-point hedonic scale) of different alcoholic beverages by men consumers (*n* = 978).

The score and loading plots of the PCA models run on alcoholic beverage liking for women (*n* = 1353) and men (*n* = 978) are reported in Figure 1a,b and Figure 2a,b, respectively.

Considering women, the first two PCs explained 45% of the total variance, with PC1 accounting for 32% and PC2 contributing a further 13%. The score plot (Figure 1a) shows that PC1 separates the samples according to alcohol content, being whisky, grappa, and liquors, which are the drinks with the highest ethanol content, positioned in the negative part of PC1 (left of the plot) and well separated from the rest of the samples. PC2 distinguishes more traditional and bitter alcoholic beverages such as beers and wines (red and white wine, dry spumante, and blonde and dark beers), positioned in the negative part of PC2, from non-alcoholic aperitif and sweet spumante, positioned in the positive part of PC2. Other beverages characterized by a relatively mild flavor (i.e., limoncello, sparkling wine, and alcoholic aperitif) are also positioned in the upper right part of the map.

A similar pattern was seen for men (Figure 2a,b). The first two PCs accounted for 39% of total variance, with PC1 accounting for 24% and PC2 for a further 15%. In addition, in this case, the score plot (Figure 2a) shows that beverages high in alcohol content (whisky, grappa, and liquors) are positioned in the negative part of PC1, separated from the rest of the samples, whereas PC2 distinguished non-alcoholic aperitifs and sweet spumante (lower right pane) from beers and wines (upper right pane). Based on PC1 and PC2 of the loading-plots, three liking patterns for different alcoholic beverages were identified. PC3 only accounted for a further 9% of the explained variance for women and 10% for men. The analysis of correlation loadings indicated that only approximately an additional 7% of consumers in both genders accounted for more than 50% of explained variance on PC3. For this reason, this dimension was not considered in cluster analysis.

We assumed that all subjects (both women and men) positioned in the negative part of PC1 (Figure 1b and Figure 2b) were those preferring spirits and were named “spirit lovers” (women: *n* = 121; men: *n* = 158), while those in the positive part preferred beverages with less alcohol content with the distinction in “beer/wine-lovers” (women in the lower right pane of Figure 1b, *n* = 592; men in the upper right pane of Figure 2b, *n* = 439) and “mild-drink lovers” (women in the upper right pane of Figure 1b, *n* = 640; men in the lower right pane of Figure 2b, *n* = 381). With “mild-drink”, we intended those beverages that either have no ethanol content (i.e., non-alcoholic aperitif) or contain alcohol but have an intense sweet taste (i.e., sweet spumante, passito wine, limoncello, and alcoholic aperitif). We assumed that these beverages elicit milder oral sensations than spirits, beer, and wine because of the reported cross-modal interaction of sweet taste on the perceived intensity of bitterness and of aversive sensations elicited by ethanol at high concentration [8].

The “spirit lovers” liked all the spirits more than the other segments, while “beer/wine lovers” liked beer (dark and blond), wine (white and red, sparkling, passito), and dry-spumante more than the other segments (see Supplementary Figure S1). The “mild-drink lovers” liked sweet spumante and alcohol-free aperitif more than the other segments, while they liked less all the other alcoholic beverages. The liking patterns of the segments were similar in men and women with only small differences. Segments did not differ in liking of the alcoholic aperitif (in men and women) and limoncello (only in men).

### 3.4. Characterization of the Three Alcoholic Beverage Liking Patterns

#### 3.4.1. Age, Gender, and Consumption of Alcoholic Beverages

Considering both genders (*n* = 2331), the percentage of ODs was considerably higher in the “mild-drink lovers” (70.3%) compared to the other two segments (Table 3).

Moreover, a higher percentage of women (47.3%) and younger individuals (53.1%) was found in the “mild-drink lovers”. A higher percentage of men (16.2%) and of the middle age group (14.4%) than expected according to the Fisher exact test was found in the “spirit lovers”. No difference by gender was found in the “beer/wine lovers”, while a higher percentage of the oldest age group (53.6%) was found in this segment.

**Figure 3:**
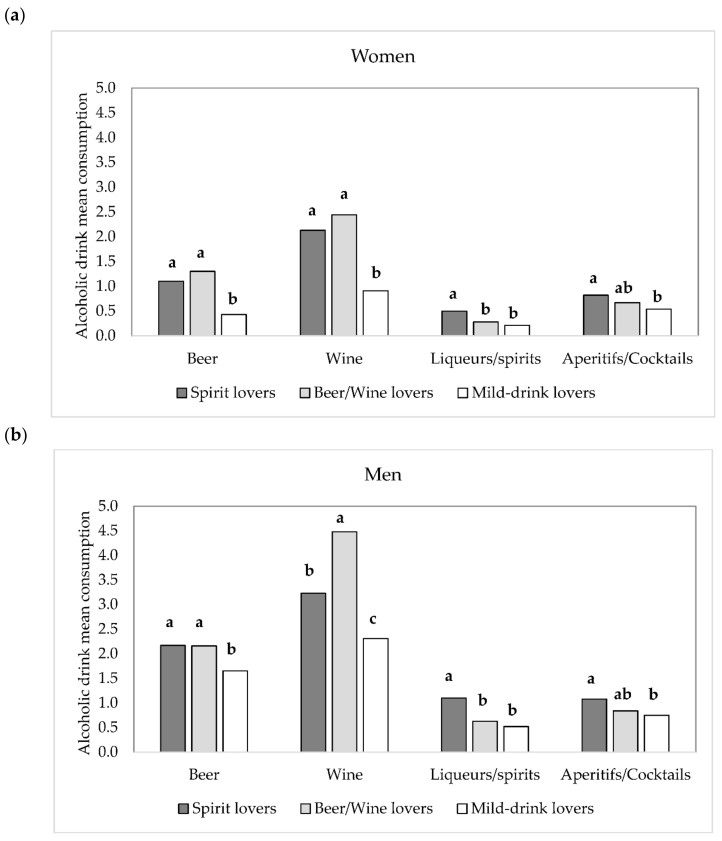
Alcoholic beverage mean consumption for the three consumers segments (spirit lovers, beer/wine lovers, and mild-drink lovers) of women, *n* = 1353 (**a**) and men, *n* = 978 (**b**). Different letters indicate significant differences according to Bonferroni post-hoc test. Mean consumption: Beer = cans/glasses per week, wine = glasses per week, spirits = times per week, alcoholic beverages = times per week.

The three segments differed in alcoholic beverage consumption (Figure 3a,b).

For both genders, spirits were consumed more by “spirit lovers”, beer by both “spirit lovers” and “beer/wine lovers”, while for aperitifs/cocktails “spirit lovers” had a higher consumption than “mild-drink lovers”. In women, wine consumption was higher in both “spirit lovers” and “beer/wine lovers”, while in men “beer/wine lovers” consumed significantly more wine than the other groups.

#### 3.4.2. Chemosensory Responsiveness

**Figure 4:**
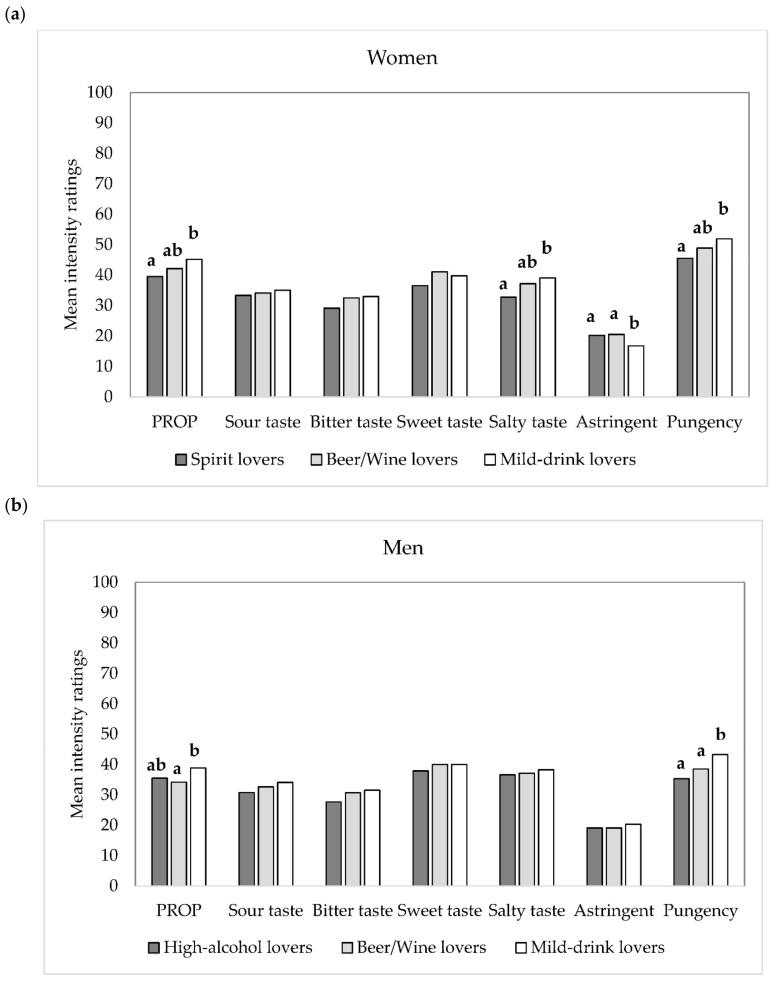
Mean intensity ratings for oral stimuli in the three consumers segments (spirit lovers, beer/wine lovers, and mild-drink lovers) of women, *n* = 1353 (**a**) and men, *n* = 978 (**b**). Different letters indicate significant differences according to Bonferroni post-hoc test.

The three segments differed significantly for their responsiveness to oral stimuli (Figure 4a,b). For women, “mild-drink lovers” perceived PROP (F_2,1350_ = 3.06; *p* = 0.047), salty taste (F_2,1350_ = 4.71; *p* = 0.009), and pungency (F_2,1346_ = 5.01; *p* = 0.007) as significantly more intense than the “spirit lovers”, but had a lower responsiveness to astringency (F_2,1341_ = 7.24; *p* < 0.001). For men, these differences were observed only for PROP (F_2,975_ = 3.62; *p* = 0.027) and pungency (F_2,968_ = 10.28; *p* < 0.0001), with “mild-drink lovers” perceiving PROP bitterness more intensely than “beer/wine lovers”, and pungency more than both groups.

## 4. Discussion

The present study, conducted on a large and representative sample of the Italian population, confirmed that demographic variables such as gender and age greatly influence both the preference and consumption of most of the alcoholic beverages considered, and that chemosensory perception may play an important role in modulating alcohol behavior.

Occasional drinkers, defined as those declaring a zero weekly consumption of beer, wine, spirits/liquors, and cocktails, represented up to 20% of the population sample. This group consisted mainly of women, with no difference according to age.

For both genders, the higher consumption of alcohol corresponded to wine and beer, while spirits and cocktails were less consumed. These results are in line with previous research and WHO data reporting wine as the most popular alcoholic drink in Italy [27], followed by beer and spirits [2]. This outcome was expected since Italy is known to be a country dominating global wine production and trade and is also considered an important reference for wine consumption linked to gastronomy [28].

In our population sample, women liked and consumed fewer alcoholic beverages than men. In general, women consumed wine and beer 2 times and spirits 2.5 times less than men. Although evidence for convergence has been observed [5], the gender gap in alcohol consumption is a common finding in several countries [2,29] and it is partially explained by different drinking norms set for men [30] and by the more aversive physiological effects alcohol may have on women [31].

We observed that alcoholic beverage consumption decreased with aging, with the exception of wine, for which a systematic increase was seen. It should be highlighted that in many contexts, drinking alcohol is socially acceptable, in particular, moderate wine consumption is considered part of well-known dietary patterns such as the Mediterranean diet [32]. Therefore, the older population might be more inclined to choose wine instead of other alcoholic beverages. Another reason for the higher wine consumption in the older group might be that alcohol consumption is strongly linked to social factors. In agreement with this assumption, sweet spumante, liquors, whisky, and both aperitifs were highly liked by young people, while red wine, passito wine, dry spumante, and grappa were more appreciated with increasing age. Interestingly, in Italy, the consumption of these latter drinks is mainly associated with meals (i.e., wine) or forms part of a ritual (e.g., grappa and after dinner fortified wines such as passito), while the drinks preferred by young people are often consumed outdoors, in the evening, during the weekend, and in more social settings (e.g., discos and dance clubs) [33]. Therefore, the different liking patterns observed in the present study may reflect a difference between generations in alcoholic beverage conceptualisation: older people associate alcohol with relaxation and may link its consumption more to a symbolic ritual, e.g., having a drink to demarcate the end of a working day [34], while young consumers may perceive alcohol more as a way to socialize and get out of routine [33].

The adoption of a within-gender approach [35] allowed pointing out some similarities between the two genders. In fact, while women liked and consumed fewer alcoholic beverages than men, when analyzed separately, similar liking patterns between genders were found. These three distinct liking patterns were largely driven by the orosensory properties of the beverages. Only a small percentage of the population sample liked spirits (“spirit lovers”), while most of the consumers liked alcoholic beverages with a lower alcohol content. Among these, two clusters have been identified, one preferring beers and wines (“beer/wine lovers”), which are notoriously characterized by sharp orosensory properties such as bitterness, sourness, and astringency [4], and one preferring non-alcoholic drinks and alcoholic drinks with intense sweet taste and/or mixers that moderate the perception of ethanol (“mild-drink lovers”) [8]. However, gender, as well as age group distribution, were considerably different within the three consumers’ segments. “Mild-drink lovers” were more likely to be women and young, “spirit lovers” were mainly men aged 30–45, while “beer/wine lovers” were the oldest group with no significant difference by gender. The three clusters differed in the consumption of alcoholic drinks and in the response to oral sensations. “Mild-drink lovers” had the highest proportion of ODs, a lower consumption of all alcoholic beverages, especially beer and wine, and were more sensitive to PROP and pungency. “Mild-drink lovers” women were also more and less sensitive to salty taste and astringency, respectively. Interestingly, there are similarities between our study and another one conducted on a large sample of American wine consumers. Although the study was performed considering different wine styles and not a variety of alcoholic beverages, Pickering et al. [16] identified a cluster of “sweet wine likers” who were mainly women, young, consumed less wine, and had a higher PROP responsiveness, as we found for “mild-drink lovers”. This convergence of findings is interesting and indicates that the characteristics of alcoholic beverages consumers may extend beyond a single cultural context and a specific beverage category. Our findings are also in line with previous research, indicating that most responsive subjects tend to drink less [8,14] and that increased perception of PROP bitterness associates with higher perception of saltiness [36,37], pungency [38,39,40,41], and greater irritation from ethanol [39,42] in water solutions, as well as with more intense perceived bitterness in alcoholic beverages [43]. In the present study, we did not measure responsiveness to irritation from ethanol but from capsaicin (pungency). Capsaicin perception is mediated by the TRPV1 ion channel, a nociceptor associated with the perception of burning, whose response is elicited and potentiated by ethanol [44,45]. Recent evidence has shown that individuals who consume beer, wine, and spirits less frequently perceive greater bitterness and burning/tingling from ethanol [7]; thus, the higher perception of these aversive sensations might be one potential reason for the disliking and lower consumption of alcoholic beverages [4]. The causality of the association between oral responsiveness and alcohol consumption is still not well understood. The intensity with which drinkers perceive taste could potentially affect their alcoholic beverage choices and drinking habits; conversely, alcohol consumption may impair taste sensitivity [15].

An unexpected finding was the reduced responsiveness to astringency by women “mild-drink lovers”. We would have expected, rather, that this group had an increased perception of astringency that may act as hindrance to the consumption of beverages, mainly wine and beer, rich in compounds eliciting this mouthfeel sensation. In fact, Thibodeau et al. [8] recently reported that individuals most responsive to sourness and astringency drank less alcohol. Astringency is an oral sensation difficult to detect and recognize [46,47], probably even more in subjects that usually do not drink alcoholic beverages characterized by this sensation. The perception of other sensations such as sweetness, sourness, and bitterness from caffeine did not differ between the consumer segments. These findings suggest that innately pleasant sensations (i.e., sweetness), and stimuli that are not elicited by ethanol (i.e., sourness) may have little influence on alcohol consumption behaviors, while the null result for bitterness responsiveness may be due to the use of caffeine, which activates different bitter receptors (TAS2R7, TAS2R10, TAS2R14, TAS2R43, and TAS2R46) [48] compared to ethanol (TAS2R13 and TAS2R38) [44]. In this context, Roura et al. [49] reported that the intensity of different bitter compounds is associated if they elicit the same TAS2R receptors. It should also be considered that several factors are involved in the preference and consumption of alcoholic beverages; therefore, it is reasonable that the perceptual dimension plays only part of the role. As a final remark, however, although differences in perception between the consumer groups were generally modest and not always significant, they were observed for almost all oral sensations and for both genders, suggesting a robust effect.

Further factors may affect liking and consumption of alcoholic beverages that were not considered in this study, related to the persons such as personality traits as sensitivity to reward [50] and sensation seeking [51], but also related to the product such as emotions and context [52]. Taken together, these factors may contribute to a deeper understanding of individual differences in liking and consumption of alcoholic beverages.

## 5. Conclusions

Large scale studies are important to confirm or refute trends observed in smaller lab-based studies. In this context, our findings confirmed and expanded the existing knowledge on the interplay between age, gender, chemosensory responsiveness, and alcohol consumption. We confirmed the importance of gender and age in alcoholic beverage liking and intake. Notwithstanding the large gender-related differences, three distinct liking patterns for different alcoholic beverages were identified in a similar way in men and women, reflecting plausible market segments: One small segment is oriented toward spirits, one toward beer/wine, and one toward drinks with mild sensory properties. Finally, we confirmed that preference and consumption of alcoholic beverages may be modulated by chemosensory responsiveness. People with higher response to oral sensations (mainly PROP bitterness and burning sensation) may experience the aversive sensations more strongly, leading to lower liking and, thus, consumption of alcohol. The systematic study of the interplay between chemosensory perception and alcohol consumption is rather new. Further studies are encouraged to better understand the causality of this association.

## Figures and Tables

**Table 1:** Effects of gender and age on mean consumption of alcoholic beverages. Different superscripts indicate significant differences according to Bonferroni post-hoc test.

Mean Consumption ^1^		Gender			Age Groups (Years)			
	Total (*n* = 2388)	*p*-Value	Women (*n* = 1390)	Men (*n* = 998)	*p*-value	18–29 (*n* = 915)	30–45 (*n* = 678)	46–60 (*n* = 795)
Beer	1.3	<0.0001	0.84 ^a^	1.92 ^b^	<0.0001	1.70 ^c^	1.40 ^b^	1.04 ^a^
Wine	2.4	<0.0001	1.67 ^a^	3.52 ^b^	<0.0001	1.71 ^a^	2.44 ^b^	3.64 ^c^
Spirits/liquors	0.4	<0.0001	0.26 ^a^	0.64 ^b^	<0.0001	0.61 ^b^	0.42 ^a^	0.32 ^a^
Aperitifs/Cocktails	0.7	<0.0001	0.60 ^a^	0.80 ^b^	<0.0001	1.06 ^c^	0.63 ^b^	0.40 ^a^

^1^ Mean consumption: Beer = cans/glasses per week, wine = glasses per week, spirits = times per week, alcoholic beverages = times per week.

**Table 2:** Effect of gender and age on mean liking (9-point hedonic scale) of alcoholic beverages. Different superscripts indicate significant differences according to Bonferroni post-hoc test (n.s. = not significant).

Drink	Gender			Age Groups (Years)			
	*p*-value	Women (*n* = 1390)	Men (*n* = 998)	*p*-value	18–29 (*n* = 915)	30–45 (*n* = 678)	46–60 (*n* = 795)
Blonde beer	< 0.0001	6.2 ^a^	7.2 ^b^	n.s.	6.7	6.8	6.7
Dark beer	< 0.0001	5.4 ^a^	6.4 ^b^	n.s.	5.9	5.8	5.9
Dry spumante	< 0.0001	5.5 ^a^	6.3 ^b^	< 0.01	5.8 ^a^	5.8 ^a^	6.2 ^b^
Sweet spumante	n.s.	5.9	6.0	< 0.0001	6.4 ^b^	5.8 ^a^	5.6 ^a^
Red wine	< 0.0001	6.4 ^a^	7.4 ^b^	< 0.0001	6.6 ^a^	7.0 ^b^	7.1 ^b^
White wine	< 0.0001	6.0 ^a^	6.7 ^b^	n.s.	6.3	6.5	6.5
Sparkling white wine	< 0.01	6.2 ^a^	6.5 ^b^	n.s.	6.4	6.2	6.4
Passito wine	< 0.0001	6.0 ^a^	6.7 ^b^	< 0.0001	6.1 ^a^	6.4 ^ab^	6.6 ^b^
Limoncello	< 0.0001	5.6 ^a^	6.3 ^b^	n.s.	6.0	6.0	5.9
Grappa	< 0.0001	3.4 ^a^	5.3 ^b^	< 0.0001	3.9 ^a^	4.5 ^b^	4.7 ^b^
Liquors	< 0.0001	4.4 ^a^	5.9 ^b^	< 0.0001	5.3 ^b^	5.3 ^b^	4.9 ^a^
Whisky	< 0.0001	3.1 ^a^	5.0 ^b^	< 0.05	4.2 ^b^	4.0 ^ab^	3.9 ^a^
Alcoholic aperitifs	*p* < 0.0001	5.9 ^a^	6.4 ^b^	*p* < 0.0001	6.8 ^c^	6.1 ^b^	5.4 ^a^
Non-alcoholic aperitifs	*p* < 0.01	6.4 ^b^	6.2 ^a^	*p* < 0.0001	6.6 ^b^	6.2 ^b^	6.1 ^a^

**Table 3:** Characterization of the three consumer segments (spirit lovers, beer/wine lovers, and mild-drink lovers) according to drinking behavior, gender, and age group. Values are reported in % with respect to the overall group (*n* = 2331).

Segments	Drinking Behavior		Gender			Age Group (Years)			
	ODs ^1^	χ^2^, *p*-Value	Women	Men	χ^2^, *p*-Value	18–29	30–45	46–60	χ^2^, *p*-Value
Spirit lovers (*n* = 279)	10.8	173.5 < 0.0001	<8.9	>16.2	33.9 < 0.0001	11.4	>14.4	10.6	65.8, < 0.0001
Beer/wine lovers (*n* = 1031)	<18.9		43.8	44.9		<35.6	45.0	>53.6	
Mild-drink lovers (*n* = 1021)	>70.3		>47.3	<39.0		>53.1	40.6	<35.8	

^1^ ODs: Occasional drinkers, respondents declaring a zero weekly consumption of beer, wine, spirits, and cocktails. < > indicate a significance per cell according to Fisher exact test.

## Supplementary Materials

The following are available online at https://www.mdpi.com/2304-8158/9/8/1131/s1. Figure S1: Mean liking scores for spirits, beers/wine and cocktails provided by the three consumers clusters (Spirit lovers, Beer/Wine lovers and Mild-drink lovers) by gender.

## References

[1] Silva A.P., Jager G., Van Zyl H., Voss H.P., Pintado M., Hogg T., De Graaf C. (2017). Cheers, proost, saúde: Cultural, contextual and psychological factors of wine and beer consumption in Portugal and in the Netherlands. Crit. Rev. Food Sci. Nutr..

[2] World Health Organization (2018). Global Status Report on Alcohol and Health 2018.

[3] Nolen-Hoeksema S. (2004). Gender differences in risk factors and consequences for alcohol use and problems. Clin. Psychol. Rev..

[4] Thibodeau M., Pickering G.J. (2019). The role of taste in alcohol preference, consumption and risk behavior. Crit. Rev. Food Sci. Nutr..

[5] Bratberg G.H., Wilsnack S.J., Wilsnack R., Haugland S.H., Krokstad S., Sund E.R., Bjørngaard J.H. (2016). Gender differences and gender convergence in alcohol use over the past three decades (1984–2008), The HUNT Study, Norway. BMC Public Health.

[6] Mäkelä P., Gmel G., Grittner U., Kuendig H., Kuntsche S., Bloomfield K., Room R. (2006). Drinking patterns and their gender differences in Europe. Alcohol Alcohol..

[7] Nolden A.A., Hayes J.E. (2015). Perceptual qualities of ethanol depend on concentration, and variation in these percepts associates with drinking frequency. Chemosens. Percept..

[8] Thibodeau M., Bajec M.R., Pickering G.J. (2017). Orosensory responsiveness and alcohol behaviour. Physiol. Behav..

[9] Bachmanov A.A., Kiefer S.W., Molina J.C., Tordoff M.G., Duffy V.B., Bartoshuk L.M., Mennella J.A. (2003). Chemosensory factors influencing alcohol perception, preferences, and consumption. Alcohol. Clin. Exp. Res..

[10] Duffy V.B., Davidson A.C., Kidd J.R., Kidd K.K., Speed W.C., Pakstis A.J., Reed D.R., Snyder D.J., Bartoshuk L.M. (2004). Bitter receptor gene (TAS2R38), 6-n-propylthiouracil (PROP) bitterness and alcohol intake. Alcohol. Clin. Exp. Res..

[11] Duffy V.B., Peterson J.M., Bartoshuk L.M. (2004). Associations between taste genetics, oral sensations and alcohol intake. Physiol. Behav..

[12] Intranuovo L.R., Powers A.S. (1998). The perceived bitterness of beer and 6-n-propylthiouracil (PROP) sensitivity. Ann. N. Y. Acad. Sci..

[13] Guinard J.-X., Zoumas-Morse C., Dietz J., Goldberg S., Holz M., Heck E., Amoros A. (1996). Does consumption of beer, alcohol, and bitter substances affect bitterness perception?. Physiol. Behav..

[14] Fischer M.E., Cruickshanks K.J., Schubert C.R., Pinto A., Klein B.E.K., Klein R., Nieto F.J., Pankow J.S., Huang G.-H., Snyder D.J. (2013). Taste intensity in the Beaver Dam offspring study. Laryngoscope.

[15] Ng G.I., Chen C.M., Graubard B.I., Hoffman H.J., Breslow R.A. (2019). Alcohol and taste intensity. Chemosens. Percept..

[16] Pickering G.J., Jain A.K., Bezawada R. (2014). Segmentation and drivers of wine liking and consumption in US wine consumers. Int. J. Wine Res..

[17] Monteleone E., Spinelli S., Dinnella C., Endrizzi I., Laureati M., Pagliarini E., Tesini F. (2017). Exploring influences on food choice in a large population sample: The Italian Taste project. Food Qual. Prefer..

[18] Peryam D.R., Pilgrim F.J. (1957). Hedonic scale method of measuring food preferences. Food Technol..

[19] Bartoshuk L.M., Duffy V.B., Green B.G., Hoffman H.J., Ko C.-W., Lucchina L.A., Marks L.E., Snyder D.J., Weiffenbach J.M. (2004). Valid across-group comparisons with labeled scales: The gLMS versus magnitude matching. Physiol. Behav..

[20] Feeney E.L., Hayes J.E. (2014). Regional differences in suprathreshold intensity for bitter and umami stimuli. Chemosens. Percept..

[21] Hayes J.E., Sullivan B.S., Duffy V.B. (2010). Explaining variability in sodium intake through oral sensory phenotype, salt sensation and liking. Physiol. Behav..

[22] Masi C., Dinnella C., Monteleone E., Prescott J. (2015). The impact of individual variations in taste sensitivity on coffee perceptions and preferences. Physiol. Behav..

[23] Bartoshuk L.M. (2000). Comparing sensory experiences across individuals: Recent psychophysical advances illuminate genetic variation in taste perception. Chem. Senses.

[24] Green B.G., Dalton P., Cowart B., Shaffer G., Rankin K., Higgins J. (1996). Evaluating the ‘‘Labeled Magnitude Scale” for measuring sensations of taste and smell. Chem. Senses.

[25] Green B.G., Shaffer G.S., Gilmore M.M. (1993). Derivation and evaluation of a semantic scale of oral sensation magnitude with apparent ratio properties. Chem. Senses.

[26] Prescott J., Soo J., Campbell H., Roberts C. (2004). Responses of PROP taster groups to variations in sensory qualities within foods and beverages. Physiol. Behav..

[27] Sluik D., Jankovic N., O’Doherty M.G., Geelen A., Schöttker B., Rolandsson O., Jong J.C.K.-D., Ferrières J., Bamia C., Fransen H.P. (2016). Alcoholic Beverage Preference and Dietary Habits in Elderly across Europe: Analyses within the Consortium on Health and Ageing: Network of Cohorts in Europe and the United States (CHANCES) Project. PLoS ONE.

[28] Rodríguez-Donate M.C., Romero-Rodríguez M.E., Cano-Fernández V.J., Guirao-Pérez G. (2019). Analysis of heterogeneity in the preferences of wine consumption. Wine Econ. Policy.

[29] Wilsnack S.C., Wilsnack R.W., Kantor L.W. (2014). Focus on women and the costs of alcohol use. Alcohol Res. Health.

[30] Chapman N.G., Nanney M., Lellock S.J., Mikles-Schluterman J. (2018). Bottling gender: Accomplishing gender through craft beer consumption. Food Cult. Soc..

[31] Thomasson H.R. (2002). Gender differences in alcohol metabolism. Recent Developments in Alcoholism.

[32] Predieri S., Sinesio F., Monteleone E., Spinelli S., Cianciabella M., Daniele G.M., Dinnella C., Gasperi F., Endrizzi I., Torri L. (2020). Gender, Age, Geographical Area, Food Neophobia and Their Relationships with the Adherence to the Mediterranean Diet: New Insights from a Large Population Cross-Sectional Study. Nutrients.

[33] Marinelli N., Fabbrizzi S., Alampi Sottini V., Sacchelli S., Bernetti I., Menghini S. (2014). Generation Y, wine and alcohol. A semantic differential approach to consumption analysis in Tuscany. Appetite.

[34] Bastian S.E.P., Danner L., Niimi J., Ristic R., Johnson T.E., Meiselman H.L. (2019). Alcoholic beverages in context. Context: The Effects of Environment on Product Design and Evaluation.

[35] Spinelli S., Dinnella C., Tesini F., Bendini A., Braghieri A., Proserpio C., Torri L., Miele N.A., Aprea E., Mazzaglia A. (2020). Gender Differences in Fat-Rich Meat Choice: Influence of Personality and Attitudes. Nutrients.

[36] Bajec M.R., Pickering G.J. (2008). Thermal taste, PROP responsiveness, and perception of oral sensations. Physiol. Behav..

[37] Fischer M.E., Cruickshanks K.J., Pankow J.S., Pankratz N., Schubert C.R., Huang G.-H., Klein B.E.K., Klein R., Pinto A. (2014). The associations between 6-n-propylthiouracil (PROP) intensity and taste intensities differ by TAS2R38 haplotype. J. Nutr. Nutr..

[38] Karrer T., Bartoshuk L. (1991). Capsaicin desensitization and recovery on the human tongue. Physiol. Behav..

[39] Prescott J., Swain-Campbell N. (2000). Responses to repeated oral irritation by capsaicin, cinnamaldehyde and ethanol in prop tasters and nontasters. Chem. Senses.

[40] Tepper B.J., Nurse R.J. (1997). Fat perception is related to PROP taster status. Physiol. Behav..

[41] Spinelli S., De Toffoli A., Dinnella C., Laureati M., Pagliarini E., Bendini A., Braghieri A., Gallina T., Sinesio F., Torri L. (2018). Personality traits and gender influence liking and choice of food pungency. Food Qual. Prefer..

[42] Bartoshuk L., Conner E., Grubin D., Karrer T., Kochenbach K., Palcso M., Snow D., Pelchat M., Danowski S. (1993). Prop supertasters and the perception of ethyl alcohol. Fifteenth Annual Meeting of the Association for Chemoreception Sciences (AChemS XV). Chem. Senses.

[43] Pickering G.J., Simukova K., DiBattista D. (2004). Intensity of taste and astringency sensations elicited by red wines is associated with sensitivity to PROP (6-n-propylthiouracil). Food Qual. Prefer..

[44] Allen A.L., McGeary J.E., Hayes J.E. (2014). Polymorphisms in TRPV1 and TAS2Rs associate with sensations from sampled ethanol. Alcohol Clin. Exp. Res..

[45] Trevisani M., Smart D., Gunthorpe M.J., Tognetto M., Barbieri M., Campi B., Amadesi S., Gray J., Jerman J.C., Brough S.J. (2002). Ethanol elicits and potentiates nociceptor responses via the vanilloid receptor-1. Nat. Neurosci..

[46] Bajec M.R., Pickering G.J. (2008). Astringency: Mechanisms and perception. Crit. Rev. Food Sci. Nutr..

[47] Ma W., Guo A., Zhang Y., Wang H., Liu Y., Li H. (2014). A review on astringency and bitterness perception of tannins in wine. Trends Food Sci. Technol..

[48] Meyerhof W., Batram C., Kuhn C., Brockhoff A., Chudoba E., Bufe B., Appendino G., Behrens M. (2010). The molecular receptive ranges of human TAS2R bitter taste receptors. Chem. Senses.

[49] Roura E., Aldayyani A., Thavaraj P., Prakash S., Greenway D., Thomas W.G., Meyerhof W., Roudnitzky N., Foster S.R. (2015). Variability in human bitter taste sensitivity to chemically diverse compounds can be accounted for by differential TAS2R activation. Chem. Senses.

[50] Tapper K., Baker L., Jiga-Boy G., Haddock G., Maio G. (2015). Sensitivity to reward and punishment: Associations with diet, alcohol consumption, and smoking. Pers. Ind. Diff..

[51] Terasaki M., Imada S. (1988). Sensation seeking and food preferences. Pers. Ind. Diff..

[52] Pierguidi L., Spinelli S., Dinnella C., Prescott J., Monteleone E. (2020). Liking patterns moderate the relationship between sensory, emotional and context appropriateness profiles: Evidences from a Global Profile study on alcoholic cocktails. Food Qual. Prefer..

