# Supplementary material for: Profiling Individual Differences in Alcoholic Beverage Preference and Consumption: New Insights from a Large-Scale Study
Source: Foods. 2020 Aug 17;9(8):1131. doi: 10.3390/foods9081131 (PMC7466201; doi:10.3390/foods9081131)
Supplement: Supplementary file 1 [file foods-09-01131-s001.pdf]

a)

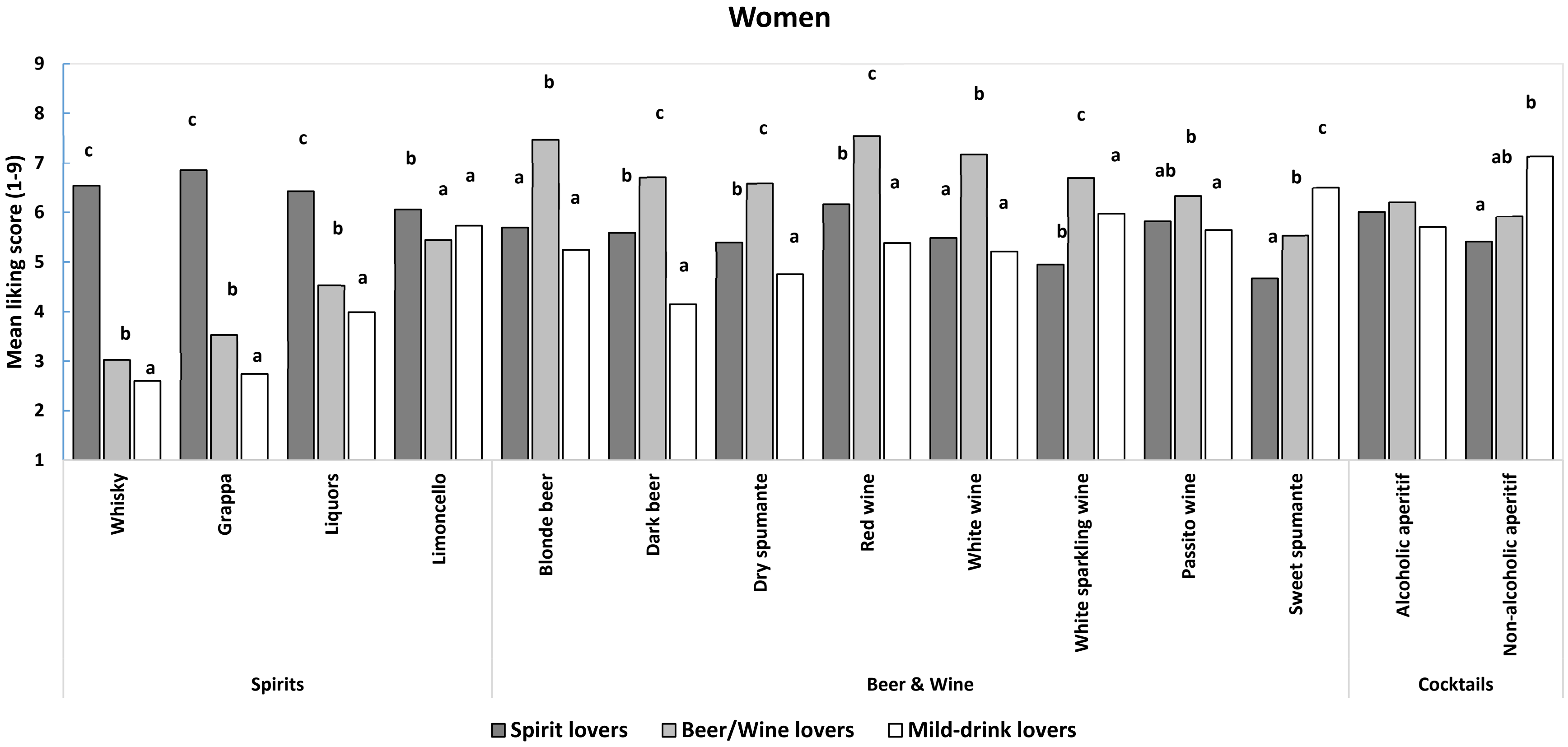

Women
Mean liking score (1-9)
9
8
7
6
5
4
3
2
1
Whisky
Grappa
Liquors
Limoncello
Blonde beer
Dark beer
Dry spumante
Red wine
White wine
White sparkling wine
Passito wine
Sweet spumante
Alcoholic aperitif
Non-alcoholic aperitif
Spirits
Beer & Wine
Cocktails
Spirit lovers
Beer/Wine lovers
Mild-drink lovers

b)

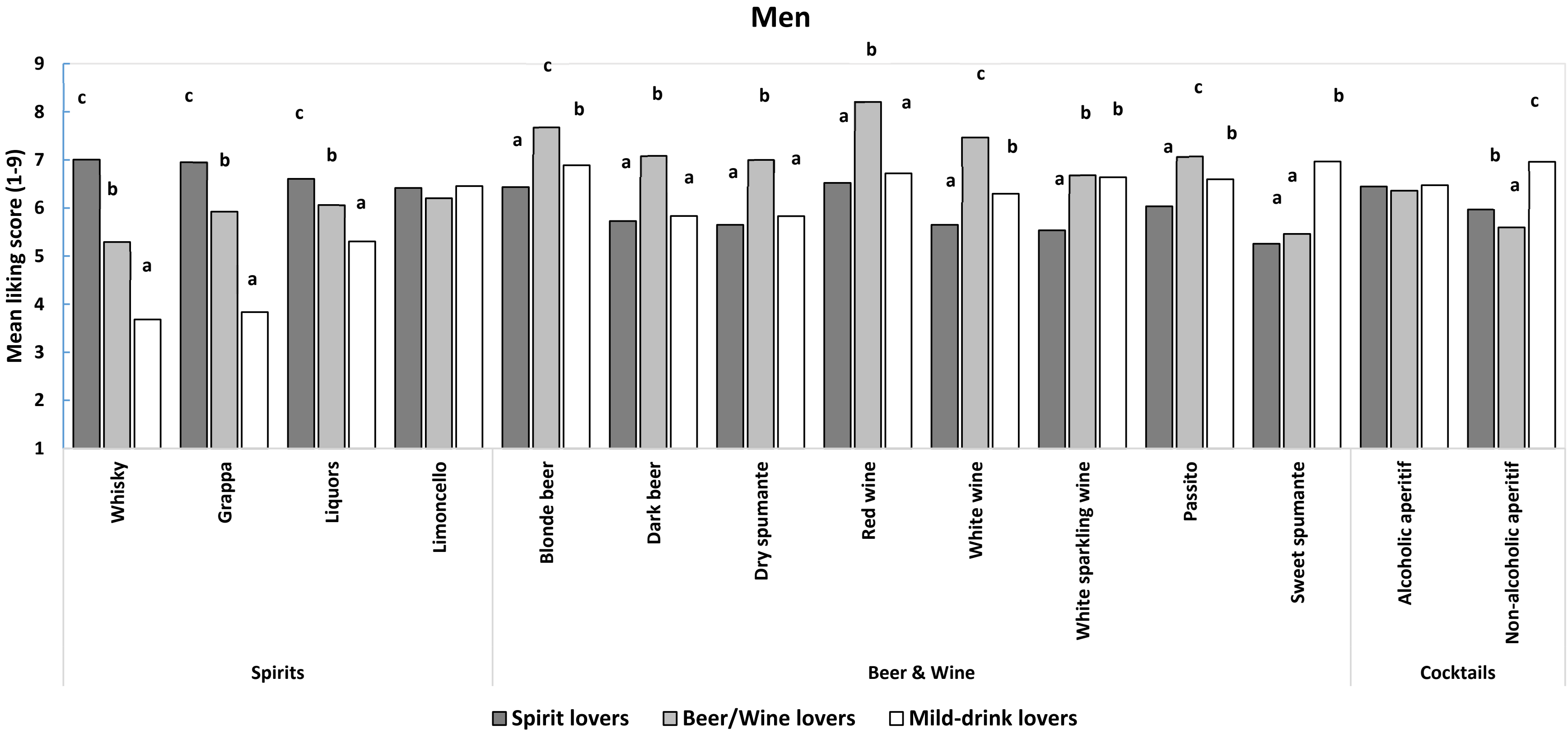

Figure S1. Mean liking scores for spirits, beers/wine and cocktails provided by the three consumers clusters (Spirit lovers, Beer/Wine lovers and Mild-drink lovers) of women (a) and men (b). Different letters indicate significant differences by beverage according to Bonferroni post-hoc test
